# Evaluating Pain, Onset of Action, Duration, and Anesthetic Efficacy of Conventional and Buffered Lidocaine in Infiltration Anesthesia: A Comparative Clinical Study

**DOI:** 10.4317/jced.63093

**Published:** 2025-10-17

**Authors:** Shehab A. Hamad, Alah D. Al-Dawoody, Ahmed Sh. Alraad, Osamah S. Ahmed

**Affiliations:** 1 BDS, MSc, MOMSRCPS (Glasg.), MFDTRCSED, FIBMS, FFDRCSI (OSOM), FDSRCPS (Glasg.), FDSRCS (Ad eundem), FICD, Professor and Consultant of Oral and Maxillofacial Surgery, Faculty of Dental Specialties, Kurdistan Higher Council of Medical Specialties, 44001, Erbil, Iraq; 2 BDS, MSc, PhD, MFDRCSI, MFDSRCPS, Assistant Professor of Orthodontics, Department of Pedodontics, Orthodontics and Preventive Dentistry, College of Dentistry, University of Mosul, 41001, Mosul, Iraq; 3 MBBS. Trainee Doctor, Palliative Care Unit. St. Rocco;s Hospice-NHS, WA5 0BW , Warrington, UK; 4 Final Year Medical student. Carol Davila University of Medicine and Pharmacy, 020022, Bucharest, Romania

## Abstract

**Background:**

Buffered local anesthetics are proposed to alleviate injection pain, decrease onset time, and extend the duration of anesthesia. This research sought to evaluate the clinical effectiveness of buffered lidocaine compared to traditional lidocaine in patients having maxillary posterior teeth extraction due to chronic periapical lesions.

**Material and Methods:**

This double-blind, prospective, randomized clinical trial involved 100 adult participants (ASA I or II), aged 18 to 60, who needed extraction of maxillary posterior teeth. Participants were randomly assigned into two equal groups: Group A was given standard 2% lidocaine with 1:100,000 adrenaline; Group B was administered buffered 2% lidocaine with 1:100,000 epinephrine combined with 8.4% sodium bicarbonate. Standardized supraperiosteal infiltrative anesthesia was given. Discomfort from the injection was assessed with the Visual Analogue Scale (VAS), and the onset and duration of anesthesia were noted as well. All surgeries were carried out by a surgeon who was unaware of the study, and results evaluated by a second investigator also blinded to the details.

**Results:**

Group B (buffered lidocaine) showed considerably reduced pain scores during injection (VAS 2.8 ± 0.7) in contrast to Group A (4.2 ± 0.9; p &lt; 0.01). The initiation of anesthesia occurred notably quicker in Group B (2.3 minutes compared to 4.7 minutes; p &lt; 0.01). The duration of anesthesia in Group B was notably greater (45.8 ± 7.6 minutes compared to 36.4 ± 8.2 minutes; p &lt; 0.01). The requirement for reinjection was not notably different among the groups (p = 0.678).

**Conclusions:**

Buffered lidocaine offers better anesthetic efficacy than standard lidocaine regarding injection comfort, quicker onset, and extended duration, making it a more effective choice for dental extractions.

## Introduction

Local anesthesia is an essential component of dental procedures, allowing for pain-free interventions. Pain associated with dental injections is a major reason why some patients avoid dental visits and cancel dental appointments. Fear about dental discomfort and anxiety may result in canceled dental appointments, which can adversely affect oral health and general well-being (1). It is important to manage pain and associated anxiety when using local anesthesia and, therefore, practitioners should take note of the anesthetic agents and application techniques, including the drugs onset, duration, and potency (2). Painless and comfortable dental and oral surgical procedures mainly depend on the constituents of the administered local anesthesia. Commonly, lidocaine, a member of the amide group, serves as an effective medication for managing pain during these procedures (3). Lidocaine becomes unstable at a pH of 7.9. To enhance its stability and extend its shelf life, it is therefore prepared at a more acidic pH. The resulting pH is 4.7, which is significantly lower than the physiological pH and may cause tissue irritation, potentially experienced by the patient as a stinging or burning feeling (4). The inclusion of a vasoconstrictor, adrenaline, in the available lidocaine formulation further lowers its pH, which helps to increase the duration of its anesthetic effects, reduce toxicity, and promote hemostasis (5). Buffered lidocaine, created by mixing sodium bicarbonate with lidocaine containing epinephrine, is utilized in dental anesthesia to enhance patient comfort and effectiveness. By adjusting the pH of an anesthetic formulation closer to its pKa, a greater proportion of the base form can theoretically penetrate the axon membrane. Raising the solution's pH to approximately physiological levels (around 7.4) help reduce burning during injection, minimizes tissue damage, and shortens the onset time (6). The time it takes for anesthesia to take effect is essential in dental treatments. Buffered lidocaine has demonstrated a decrease in onset time for anesthesia in certain situations, especially in inflamed areas and during inferior alveolar nerve blocks (7). Nevertheless, the practical importance of this decrease is contested, since the time needed to prepare the buffered solution could negate the advantages. Additionally, other research has indicated no notable difference in onset time between buffered and non-buffered lidocaine (8). Buffered lidocaine has also been investigated for its ability to alleviate pain during dental treatments. Certain research indicates that adding sodium bicarbonate to lidocaine may lessen the discomfort of the injection while also preserving the anesthesia's effectiveness during dental work (9). However, some studies reported no noteworthy differences in pain relief between buffered and nonbuffered lidocaine during dental extractions and other types of procedures (10). Although Lidocaine is still considered the gold standard local anesthetic worldwide and remains the anesthetic of choice in dentistry, many studies have favored the use of Articaine, whose systemic toxicity is lower, the effectiveness is higher, and the working time is longer (11 , 12). However, several studies have reported of paresthesia after the administration of IANB using Articaine (13 , 14). The precise mechanism responsible for this complication is unclear. Nevertheless, the existing literature shows that the likely reason for this reaction is an increased concentration of Articaine (4%) compared to traditionally employed 2% Lidocaine (15 , 16). This research sought to assess the pain perception, onset time, duration, and effectiveness of buffered lidocaine compared to conventional lidocaine in infiltration anesthesia for the extraction of maxillary posterior teeth. The null hypothesis posited that there would be no notable difference between buffered and conventional lidocaine regarding pain, onset, duration, and overall anesthetic effectiveness.

## Material and Methods

**Figure 1:**
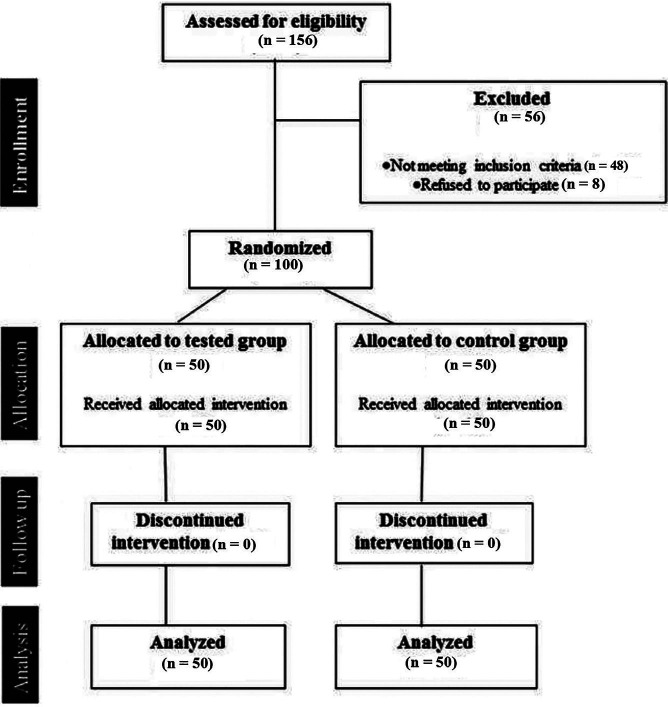
Flowchart of the study.

This randomized, prospective, double-blind clinical trial was conducted in hospital dental clinic, from September 2024 to April 2025; on patients presented for extraction of maxillary posterior teeth with chronic periapical lesions. All the procedures in accordance with the ethics standards given in the 1964 Declaration of Helsinki, as revised in 2013. Ethical approval was obtained from the Kurdistan Board of Medical Specialties (No. 440, dated 29-08-2024), and written informed consent was obtained from all participants. A total of 100 patients requiring extraction of maxillary posterior teeth with periapical lesions were enrolled. Inclusion criteria included: Age 18-60 years, ASA I or II status, no history of allergy to local anesthetics. Exclusion criteria included: Pregnant or lactating women, presence of systemic infection, patients requiring multiple extractions. Subjects taking any medication such as analgesics, narcotics, sedatives and antidepressants that may affect anaesthetic assessment Sample size was calculated using G*Power 3.1. Based on a pilot study (n=10), an effect size of 0.8 for VAS reduction (=0.05, power=90%), 50 patients per group were required. The study flowchart is presented in Fig. 1.

1

Participants were randomly assigned to one of two groups, 50 patients in each group: Group A (Control): Received conventional 2% lidocaine with 1:100,000 adrenaline. Group B (Experimental): Received buffered 2% lidocaine with 1:100,000 epinephrine Buffered solution was prepared by adding 0.18 mL of 8.4% sodium bicarbonate (NaHCO3) to 1.8 mL 2% lidocaine with 1:100,000 epinephrine (1:10 ratio) immediately before injection. The Final pH was ~7.4. The pH of conventional (A) and buffered (B) solutions was verified using a calibrated pH meter (Model XYZ, Brand). Mean pH: Group A = 4.2 ± 0.1; Group B = 7.3 ± 0.1. All patients received 1.8 mL anesthetic per tooth. Dosing was standardized; no adjustments were made for weight/age as all were ASA I/II adults. Standardized supraperiosteal infiltration anesthesia was administered using a 27-gauge needle. The needle injected the anesthetic solution without touching the bone to minimize pain. The anesthetic injection speed was always 1 mL/min. All procedures were done by a single maxillofacial surgeon who was unaware of the type of local anaesthesia given to the patient. All parameters were assessed by a second investigator, who was blinded for the study. Pain during injection, onset of action, and duration of anesthesia were recorded, as well as the need for reinjection. Baseline pain (pre-injection) was assessed via VAS for chronic periapical pain. Pain on infiltration was evaluated using the Visual Analogue Scale (VAS). The VAS has markings ranging from 0 to 10, 0 being 'no pain' and 10 being 'worst possible pain'. Patients were asked to rate their pain at the time of infiltration based on the intensity of pain experienced. The onset of local anaesthesia is defined as the first sensation of numbness or tingling in the anaesthetised region. It was calculated from the point of retrieval of the needle after the injection to the time of onset of numbness, which was demonstrated on probing. The time at which the first analgesic drug was administered after extraction was used to calculate the duration of anaesthesia (in minutes). Data were analyzed using SPSS software. Descriptive statistics summarized baseline characteristics. Independent t-tests and Mann-Whitney U tests compared continuous variables, while Chi-square tests evaluated categorical outcomes. A p-value of &lt;0.05 was considered statistically significant.

## Results

The demographic and baseline clinical characteristics of the participants are summarized in Table 1.

Table 1

Both groups were comparable in terms of age, gender distribution, and baseline pain scores. Patients in Group B reported significantly lower pain levels during injection compared to Group A (p &lt; 0.01). The mean (±SD) VAS score of group B was 2.8(±0.7) compared to 4.2(±0.9) in group A The onset of action was significantly faster in Group B (p &lt; 0.01). The mean onset time for buffered lidocaine was 2.3 minutes compared to 4.7 minutes for conventional lidocaine. The duration of anesthesia was significantly longer in Group B (p &lt; 0.01). The mean duration for buffered lidocaine was 45.8 minutes compared to 36.4 minutes for conventional lidocaine. No significant difference was noted between the group A (8%) and group B (4%), P=0.678 (Table 2).

Table 2

## Discussion

This randomized, double-blind clinical trial demonstrates that buffered 2% lidocaine with epinephrine (Group B) significantly improves patient comfort and anesthetic efficacy compared to conventional lidocaine with adrenaline (Group A) during the extraction of maxillary posterior teeth with chronic periapical lesions. The findings align with recent advancements in local anesthetic buffering techniques while offering novel insights into the clinical benefits of pH adjustment in dental anesthesia. The significantly lower VAS scores during injection in Group B (2.8 vs. 4.2, p &lt; 0.01) corroborate recent evidence that buffering local anesthetics reduces discomfort by neutralizing their acidic pH. Lidocaine solutions typically have a pH of 3.5-5.5, which can irritate tissues and activate nociceptors. Buffering with sodium bicarbonate (pH ~7.4) mitigates this effect, as demonstrated by the study of Gorrela et al., which reported a 71% reduction in injection pain with alkalinized lidocaine (17). Our results further validate this trend, emphasizing the clinical relevance of pH optimization for patient comfort. Group B exhibited a faster onset (by 2.4 minutes) and longer anesthetic duration (by 9.4 minutes), consistent with studies linking buffered solutions to enhanced pharmacodynamic profiles. The accelerated onset aligns with the hypothesis that a neutral pH facilitates faster diffusion of the uncharged lidocaine base into nerve fibers. A 2022 trial by Fernandez and Savina similarly reported a 50% reduction in onset time for buffered lidocaine in maxillary premolars extraction for orthodontic purposes (18). In the same Guto et al. (19) Buffered lidocaine demonstrated a faster onset time, with reductions of approximately 48 seconds in alveolar nerve block anesthesia. The longer duration of buffered lidocaine/adrenaline in this study was also supported by Gorrela et al. (17) who found that buffered solutions extend the duration of anesthesia by 15% (23 minutes). The prolonged duration in buffered lidocaine may stem from reduced vasodilation caused by acidic solutions, preserving epinephrine's vasoconstrictive efficacy. However, some studies indicate that while onset time improves, the duration of anesthesia may be negatively affected, with a decrease of up to 19.6 minutes noted in buffered solutions (20). Despite the advantages in pain reduction, our study coincides with studies indicate that buffered lidocaine does not significantly decrease the number of reinjections required for adequate anesthesia (21). A longer duration of anesthesia (45.8 minutes) is beneficial for complicated extractions (such as multi-rooted molars or sclerotic bone) or for patients who are anxious and need a more gradual pace during the operation. Although straightforward extractions might take less than 20 minutes, the presence of periapical pathology can extend the duration of the surgery. Despite some positive findings, several studies concluded that buffered lidocaine does not consistently outperform non-buffered alternatives in terms of overall efficacy (7 , 8). While buffering may enhance certain aspects of local anesthesia, it is essential to consider that individual patient responses can vary significantly, and some may not experience the anticipated benefits. Patient age, pain threshold, anxiety levels, and individual variability in tissue pH can influence anesthetic perception and response. Some areas (e.g., maxilla vs. mandible, inflamed vs. non-inflamed tissue) may show different responses to buffering due to vascularity and pH variations in addition to variations in injection technique, needle gauge, and volume of anesthetic used may mask the potential benefits of buffering. While articaine-containing anesthetics (e.g., 4% articaine with 1:100,000 epinephrine; pH ~4.0) offer advantages in efficacy and duration due to higher lipid solubility and thiophene ring structure (Clark &amp; Perrin equation) (22), they were not selected for this study. Buffered lidocaine remains clinically relevant in regions where articaine is unavailable, cost-prohibitive, or contraindicated (e.g., in patients with sulfite allergies common in articaine formulations). Additionally, buffering is a low-cost technique applicable to widely available lidocaine, enhancing accessibility. Electric pulp testing is useful tool for assessing pulpal vitality, predicting anesthetic effectiveness, and identify potential anesthetic failures, allowing for supplemental injections when necessary (23). However, in a study on primary maxillary molars, EPT failed to reliably assess pulpal anesthesia, as the correlation with pain response was not statistically significant (24). The application of an electric pulp tester to evaluate the effects of anesthesia in this study was not practical for two primary reasons. Firstly, the teeth selected for extraction were non-vital and had associated periapical lesions, making them inappropriate for pulp testing. Secondly, since the infiltration technique was utilized for administering anesthesia, it only affected the immediate area surrounding the injection point. Consequently, adjacent teeth could not be accurately assessed for pulp testing, as they may not have been anesthetized. On the other hand, if a nerve block technique had been used, it could have allowed for the assessment of anesthetic parameters in neighboring vital teeth within the same nerve supply area. Thus, we relied on other clinical and subjective methods to measure the onset, duration, and effectiveness of the anesthesia. This study adds robust evidence supporting buffered lidocaine's superiority in maxillary posterior teeth extractions-a region understudied in prior trials. The combination of reduced pain, faster onset, and prolonged analgesia addresses key challenges in dental anesthesia, particularly for patients with dental anxiety or complex periapical pathologies. The protocol's simplicity (on-site buffering with sodium bicarbonate) also enhances its practicality for clinical adoption. The focus on maxillary posterior teeth limits generalizability to other regions (e.g., mandible) or procedures (e.g., implants) may be considered as a limitation in this study. We used a fixed 1:10 sodium bicarbonate ratio. Future studies could explore optimized ratios or premixed formulations. While VAS is widely validated, adjunctive biomarkers (e.g., heart rate variability) could provide objective pain assessments.

## Conclusions

This trial demonstrates that buffered lidocaine with adrenaline offers significant advantages over conventional formulations in maxillary posterior tooth extractions. The findings align with contemporary trends favoring pH-adjusted anesthetics to enhance patient comfort and procedural efficiency. Future research should investigate buffering's impact in diverse dental contexts and refine protocols for broader clinical application.

## Figures and Tables

**Table 1:** Demographics and baseline clinical characteristics.

Variables	Group A (Conventional Lidocaine) N=50	Group B (Buffered Lidocaine) N=50	p-value
Mean age (years)	35.2 ± 10.4	34.8 ± 9.8	0.78
Gender (Male/Female)	28/22	26/24	0.67
Number of extracted teeth(First molar/ Second molar)	23/27	20/30	0.54
Mean duration of extraction (minutes)	3.2 ± 0.4	3.1 ±0.3	0.16
Baseline pain (VAS)	6.5 ± 1.2	6.7 ± 1.1	0.53

1

**Table 2:** Injection pain, onset and duration of anesthesia and reinjection rate.

Parameter	Group A(Conventional lidocaine)	Group B(Buffered lidocaine)	p-value
Pain during injection (VAS)	4.2 ± 0.9	2.8 ± 0.7	< 0.01
Onset time (minutes)	4.7	2.3	< 0.01
Duration of anesthesia (minutes)	36.4 ± 8.2	45.8 ± 7.6	< 0.01
Reinjections required	8% (4/50)	4% (2/50)	0.678

2

## Data Availability

The datasets used and/or analyzed during the current study are available from the corresponding author.
